# Impact of serum C-reactive protein level as a biomarker of cancer dissemination in canine lymphoid neoplasia

**DOI:** 10.14202/vetworld.2022.2810-2815

**Published:** 2022-12-10

**Authors:** Nawin Manachai, Duangchanok Umnuayyonvaree, Panitnan Punyathi, Anudep Rungsipipat, Kasem Rattanapinyopituk

**Affiliations:** 1 Department of Pathology, Faculty of Veterinary Science, Chulalongkorn University, Pathumwan, Bangkok, Thailand; 2 Center of Excellence - Companion Animal Cancer (CE-CAC), Chulalongkorn University, Pathumwan, Bangkok, Thailand; 3 Department of Companion Animals and Wildlife Clinics, Faculty of Veterinary Medicine, Chiang Mai University, Chiang Mai, Thailand

**Keywords:** advanced stage lymphoma, biomarker, dogs, serum C-reactive protein

## Abstract

**Background and Aim:**

C-reactive protein (CRP) is a highly sensitive but non-specific acute phase protein that has been widely used to predict the biological behavior of patients with cancer. This study aimed to examine the significance of the serum CRP biomarker in predicting the prognosis of dogs with lymphoma.

**Materials and Methods:**

Blood samples (5 mL) were collected from 34 lymphoma dogs and control healthy dogs. Canine lymphoma clinical staging was classified using the World Health Organization (WHO) criteria. All lymphoma dogs were reclassified into two groups based on the disease stage. Stages IV and V were designated as advanced stages, and Stages I–III were designated as other stages. The serum CRP level was then determined using a commercial canine CRP fluorescent immunoassay kit and routine hematological and biochemical analyses. C-reactive protein levels, circulating inflammatory parameters, such as neutrophil-to-lymphocyte ratio, lymphocyte-to-monocyte ratio, and platelet-to-lymphocyte ratio, and albumin levels were compared between advanced stages (IV and V) and Stages I to III using Mann–Whitney U tests. Receiver operating characteristic (ROC) curves were also generated to determine the cutoff value, diagnostic sensitivity, and specificity of the CRP level.

**Results:**

A prospective study identified 34 dogs recently diagnosed with canine lymphoma. C-reactive protein levels were significantly higher in lymphoma dogs in advanced stages (IV and V) than in lymphoma dogs in Stages I–III. According to the ROC curve analysis, a CRP cutoff level of 54.1 mg/L indicates advanced-stage canine lymphoma, which can be used as a biomarker to predict cancer dissemination.

**Conclusion:**

Serum CRP concentrations can assist clinical decision-making on the WHO stage in lymphoma dogs in clinical applications. The limitations of this study include a small number of lymphomas and no survival analysis.

## Introduction

Lymphoma is one of the most common cancers in dogs, accounting for 7%–24% of all canine tumors [1] and more than 80% of canine malignancies [2]. Lymphoma was characterized as a heterogeneous disease with various subtypes and malignancies [3]. Several prognostic factors in lymphoma dogs have been extensively studied, including World Health Organization (WHO) clinical stage, immunophenotypes, and the presence of hypercalcemia and hypoalbuminemia [2, 4]. At present, gene expression profiling studies have revealed a relationship between lymphoma and the host inflammatory response and a role for tumor microenvironment gene signatures in the clinical outcome of patients with lymphoma [5]. Multiple studies have found that the host’s inflammatory response is important in cancer metastasis and prognosis [5]. Recently, a number of inflammatory indices, including C-reactive protein (CRP), neutrophil-to-lymphocyte ratio (NLR), platelet-to-lymphocyte ratio (PLR), and modified Glasgow prognostic score, have been considered as independent prognosis factors in patients with cancer [2, 6, 7]. Moreover, serum-based and peripheral blood cell tests are routinely investigated in patients with cancer. This simple test can be used to determine the severity of the inflammatory response in patients with cancer [8].

C-reactive protein is a highly sensitive but non-specific acute phase protein (APP) produced in the liver in response to tissue injury and inflammation. C-reactive protein plays an important role in innate immunity [9]. C-reactive protein is a biomarker for human lymphoid neoplasia [2]. C-reactive protein is increased in neoplastic diseases by tissue damage and promotes the inflammatory cascade [10]. Multiple studies have shown that high serum CRP levels are associated with a poor prognosis in canine lymphoid neoplasia [11]. However, research on inflammatory markers such as CRP in conjunction with hematological profiles is still limited [12]. Moreover, the use of serum CRP to determine disseminated lymphoma in dogs has not been thoroughly investigated.

This study aimed to examine pre-treatment serum CRP levels in relation to serum albumin and other circulating inflammatory parameters in dogs with advanced-stage lymphoma. Furthermore, we aimed to determine the optimal cutoff value for serum CRP concentrations to use this as a clinically relevant biomarker for diagnosing disseminated disease.

## Materials and Methods

### Ethical approval

This study was approved by the Animal Care and Use Committee of the Faculty of Veterinary Science, Chulalongkorn University, Thailand (No. 1731053).

### Study period and location

This study was conducted from October 2020 to September 2021 at the Department of Pathology, Faculty of Veterinary Science, Chulalongkorn University, Bangkok, Thailand.

### Clinical cases

This study included 34 dogs from the Oncology Clinic, Small Animal Teaching Hospital, Faculty of Veterinary Science, Chulalongkorn University, Bangkok, who had been clinically diagnosed with canine lymphoma. The cytological or histopathological evaluation of lymph nodes or tumor-associated tissue samples obtained through fine-needle aspiration or biopsy specimens was used to make the final diagnosis of lymphoma. Routine hematological and biochemical analyses were performed to evaluate the clinicopathological factors of lymphoma dogs. Moreover, clinical staging for canine lymphoma was divided into five stages based on the WHO criteria [4, 13]. All lymphoma dogs were reclassified into two groups based on the disease stage. Stages IV and V were designated as advanced stages, and Stages I–III were designated as other stages. Patients with prior chemotherapy and systemic corticosteroid administration were excluded from the study.

### Sample collection

Blood samples (5 mL) were collected from 34 lymphoma dogs. Control samples were obtained from healthy dogs that were blood donors from the Blood Bank Centre, Chulalongkorn University. All samples were allowed to clot and then centrifuged at 1800× *g* for 10 min. The serum was separated and stored at −80°C in another plain tube for CRP determination.

### C-reactive protein measurement

The serum CRP level was determined using a commercial canine CRP fluorescent immunoassay kit (Vcheck Canine CRP, BIONOTE, Hwaseong, Korea), according to the manufacturer’s instructions. A serum CRP level of ≤10 mg/L could not be indicated.

### Statistical analysis

C-reactive protein levels, circulating inflammatory parameters, such as NRL, lymphocyte-to-monocyte ratio (LMR), and PLR, and albumin levels were compared using Mann–Whitney U tests between advanced stages (IV and V) and stages I−III. Receiver operating characteristic (ROC) curves were generated using MedCalc Version 20.118 statistical software (MedCalc Software Ltd, Ostend, Belgium)to calculate the cutoff value, diagnostic sensitivity, and specificity of CRP level to be used as a potential biomarker for assessing the advanced stage of lymphoma dogs.

## Results

### Patient characteristics

Patient characteristics included in the studies are summarized in Table-1. In this study, 34 dogs were diagnosed with lymphoma based on cytology or histopathology; 30 (88.24%) of these had multicentric form, and four dogs (11.76%) had extranodal diseases, such as alimentary, spleen, liver, and tonsil. All lymphoma dogs had a median age of 10 years (range 1–17), with 17 (50%) males and 17 (50%) females. There were 24 mixed-breed dogs and 10 pure-breed dogs. According to the WHO classification system for canine lymphomas as demonstrated by clinical staging, one dog (5.88%) was classified as Stage I, 21 dogs (61.76%) as Stage III, which represented the majority of the present study, eight dogs (23.52%) as Stage IV, and three dogs (8.82%) as Stage V. However, there were no cases of Stage II in this study. We classified Stages IV and V as the advanced stages of the disease (32.34%).

**Table-1:** Patient characteristics of 34 dogs with lymphoma.

Parameter		
Age (years)		
Mean ± SD	9.72 ± 4.27	
Median	10	

	**Number**	**Percentage**

Sex		
Female	17	50
Male	17	50
Breed		
Mixed breeds	24	70.59
Pure breeds	10	29.41
Stage		
I	2	5.88
II	0	0
III	21	61.76
IV	8	23.52
V	3	8.82
Anatomical classification		
Multicentric	30	88.24
Extranodal	4	11.76
CRP (mg/L)		
Lower than 10	7	20.59
More than 10	27	79.41

SD=Standard deviation, CRP=C-reactive protein

### Pre-treatment of serum CRP concentration

**Figure-1:**
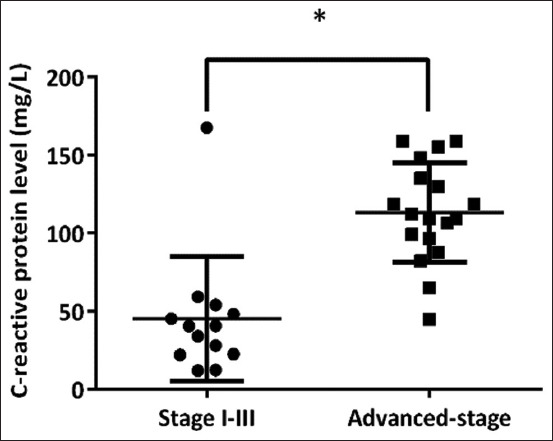
Box and whisker plot showing the serum C-reactive protein level between Stage I and III and advanced-Stage IV and V of canine lymphoma with *p < 0.01.

**Figure-2:**
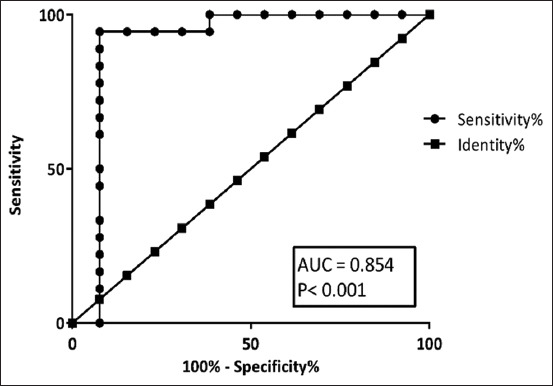
Receiver operating characteristic curve for C-reactive protein level between Stage I and III and advance-stage (Stage IV and V) of canine lymphoma, the 95% confidence intervals of the area under the curve was 0.854 with p < 0.001.

All serum of healthy control samples had a low level of CRP (≤10 mg/L). For all lymphoma dogs, the majority of 27 dogs (79.4%) had CRP concentrations >10 mg/L; however, seven dogs (20.6%) demonstrated low CRP concentrations ≤10 mg/L (Table-1). The clinical staging distributions in dogs with lymphoma that was affected by high and low levels of serum CRP are shown in Table-2. In this study, the median value for the CRP concentration in all lymphoma cases is 56.65 mg/L (Table-3). Interestingly, CRP levels are significantly higher in lymphoma dogs in the advanced stage (Stages IV and V, p = 0.00048), as indicated by the box and whisker plot (Figure-1). Moreover, we performed a ROC curve analysis to determine the CRP cut-off to differentiate the two groups aiming for the diagnostic potential of serum CRP concentrations. Receiver operating characteristic curve analysis indicated that the area under the curve (AUC) for CRP was 0.85 (Figure-2). The optimal CRP cutoff value of 54.1 mg/L had 85.71% sensitivity and 85% specificity for predicting lymphoma dogs in the advanced stages of the disease.

**Table-2:** Distribution of clinical staging affected by high (more than 10 mg/L) and low (≤10 mg/L) level of serum CRP in dogs with lymphoma.

Level of serum CRP	All lymphoma dogs (n=34)	Advanced stage (IV and V) lymphoma dogs (n=11)	Stage I – III lymphoma dogs (n=21)
Low	7	0	7
High	27	11	15

CRP=C-reactive protein

### Pre-treatment of albumin and circulating inflammatory parameters (NLR, LMR, and PLR) in lymphoma dogs

In addition to using serum CRP as an advanced-stage biomarker, albumin and circulating inflammatory parameters, such as NLR, LMR, and PLR, were investigated. The results of these biomarkers are displayed as box and whisker plots (Supplentary Figures-S1–S4). There was no significant difference between the groups in albumin and NRL, LMR, and PLR. The median value of albumin concentration and circulating inflammatory parameters with p values for each group are shown in Table-3.

**Table-3:** Median value of pre-treatment levels of serum CRP and circulating inflammatory parameters in lymphoma dogs.

Biomarkers	All cases (n = 34)	Advanced stage (IV and V)	Stage I–III	p-value
Serum CRP level	56.65	109.4	40.8	0.00048*
Serum albumin	2.6	2.25	2.7	0.019
White blood cell count	11,815	11,840	14,530	0.293
Neutrophils count	8,423	7,980	9691	0.294
Lymphocytes count	1,357	1,114	1494	0.542
Monocyte count	543	479	1,201	0.107
Platelets count	142,000	97,000	165,000	0.103
Neutrophil to lymphocyte ratio	4.9	5.47	3.7	0.395
Lymphocyte to monocyte ratio	2.12	1.97	2.09	0.379
Platelet to lymphocyte ratio	122.20	98.16	125.69	0.516

* p < 0.05, CRP=C-reactive protein

## Discussion

**Supplementary Figure-1:**
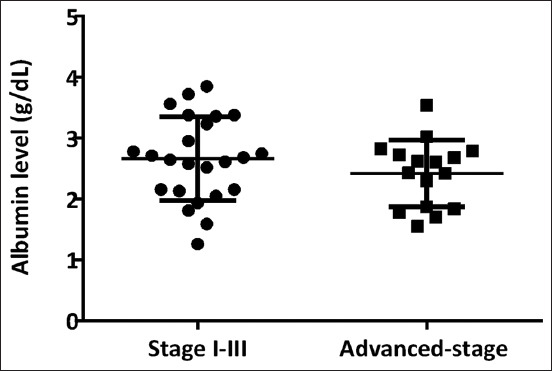
Box and whisker plot demonstrating the serum albumin concentration between Stage I and III and advanced-stage of canine lymphoma.

Tumor-promoting inflammation has been identified as a contributing factor to the current hallmarks of cancer [14]. Changes in inflammatory status in patients with cancer, as a result of changes in the tumor microenvironment and APP components, have been shown to predict the duration of remission and disease survival in a variety of solid tumors and lymphoid malignancies [11, 15]. The relationship between cancer and APP has been studied in human and veterinary medicine, with a particular focus on a positive correlation between CRP concentration and clinical manifestations of lymphoid neoplasia at the time of diagnosis [16–18]. However, advances in diagnostic assessment for dogs with disseminated lymphoma have yet to be fully investigated. In this study, the use of serum CRP was compared with pre-treatment albumin and circulating inflammatory markers in lymphoma dogs at various stages. C-reactive protein is a sensitive biomarker of inflammation in various cancers [9, 19]. It is a classical acute phase, non-specific inflammatory protein synthesized by hepatocytes, primarily under the control of interleukin-6 (IL-6) with other inflammatory cytokines. C-reactive protein elevation is believed to reduce T-lymphocyte response to tumor, which may contribute to disease progression [9]. In this study, dogs with advanced-stage (Stages IV and V) lymphoma had significantly higher CRP levels than dogs with Stages I−III lymphoma (p = 0.00048). Furthermore, the ROC curve was used to investigate the usefulness of serum CRP levels in determining disseminated disease in lymphoma dogs. The AUC indicated that serum CRP level has a good ability to differentiate between Stages I–III and advanced stages of lymphoma in dogs in ROC analysis, and 54.1 mg/L was the cutoff CRP level with the appropriate balance for sensitivity (85.71%) and specificity (85.00%). According to this finding, dogs with advanced-stage lymphoma have an increased tumor burden and enlarged lymph nodes, resulting in intralesional necrosis and localized inflammation. The tumor’s rapid growth causes high cell turnover, which is characterized by apoptosis and/or necrosis of neoplastic cells and the subsequent release of cytokines. Tumor cells can also secrete chemokines, which attract local immune cells such as neutrophils and macrophages [20, 21]. The immune cells then release cytokines into the blood, including interleukins IL-1, IL-6, and TNF-α, which stimulate hepatocytes to secrete APPs and CRP [22]. Additionally, albumin is a traditional negative APP that is also synthesized by the liver, and its levels decrease as the positive APP levels increase [23]. The median albumin concentration in lymphoma dogs in this study was 2.6 g/dL (Table-3). However, no significant difference was found between dogs with lymphoma in Stages I–III (median of 2.7 g/dL) and dogs with advanced-stage lymphoma (median of 2.25 g/dL, p = 0.019 Supplementary Figure-1, Table-3). These results suggest that the WHO stage has no effect on albumin concentration. However, serum albumin could be used to assess the nutritional status and negative nitrogen balance in malnourished patients with cancer [24].

**Supplementary Figure-2:**
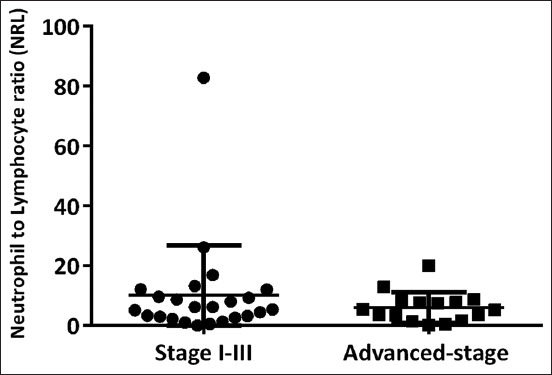
Box and whisker plot demonstrating the neutrophil to lymphocyte ratio between Stage I and III and advanced-stage of canine lymphoma.

**Supplementary Figure-3:**
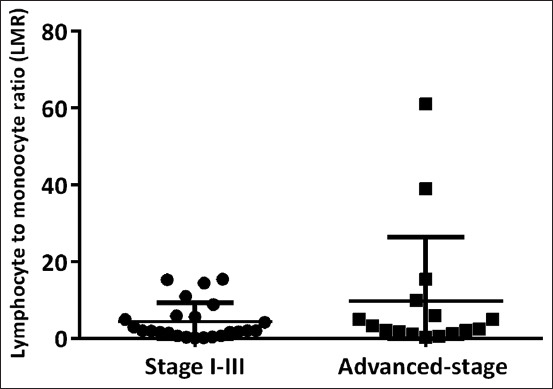
Box and whisker plot demonstrating the lymphocyte to monocyte ratio between Stage I and III and advanced-stage of canine lymphoma.

**Supplementary Figure-4:**
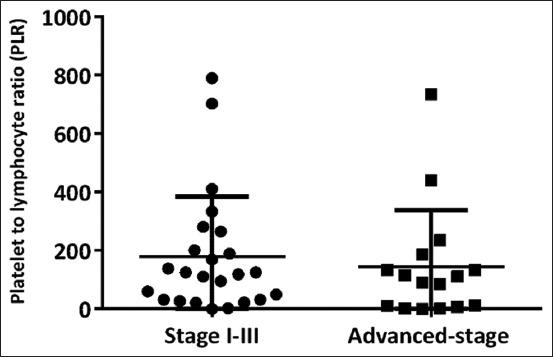
Box and whisker plot demonstrating the platelet to lymphocyte ratio between Stage I and III and advanced- stage of canine lymphoma.

Multiple studies have revealed that the ratio of various types of circulating blood cells can be used to predict lymphoma prognosis based on the relationship between inflammation and lymphoma, as demonstrated by serum CRP elevation and circulating blood cells. Neutrophil-to-lymphocyte ratio, LMR, and PLR have been studied to predict prognosis in lymphoid malignancies [8, 25]. These circulating inflammatory parameters, including NLR, LMR, and PLR, were investigated in this study. However, all circulating inflammatory markers did not differ significantly between Stages I to III and advanced-stage lymphoma (p = 0.395, p = 0.379, and p = 0.516, respectively (Supplementary Figures-2–4, Table-3). In agreement with Mutz *et al*. [26], we discovered that NLR was not a useful prognostic biomarker for canine lymphoma, possibly due to the high variation in white blood cells and platelet absolute numbers. In contrast, NLR was a good discriminatory index for screening high-grade mast cell tumor in dogs prior to histopathological evaluation [27]. These findings suggest that lymphomas are one of the most heterogeneous tumors [13].

## Conclusion

This study suggests that serum CRP concentrations have the potential in clinical applications for assisting clinical decision-making on the WHO stage in lymphoma dogs. C-reactive protein could be used as a prognostic biomarker for canine lymphoma, indicating advanced-stage and disseminated disease. There are some limitations to our study: Only a small number of canine lymphoma cases were investigated in this study, and no survival analysis was performed. Therefore, additional research should be conducted, including the addition of sample size and a survival analysis investigation.

## Authors’ Contributions

NM, KR, and AR: Contributed to the project’s conceptual design and editing of the manuscript. NM, DU, and PP: Conducted the experiment. NM: Performed the statistical analysis and drafted the manuscript. All authors have read and approved the final manuscript.
